# Obesity and atrial fibrillation: a narrative review from arrhythmogenic mechanisms to clinical significance

**DOI:** 10.1186/s12933-023-01913-5

**Published:** 2023-07-29

**Authors:** Hongyang Shu, Jia Cheng, Na Li, Zixuan Zhang, Jiali Nie, Yizhong Peng, Yan Wang, Dao Wen Wang, Ning Zhou

**Affiliations:** 1 Division of Cardiology, Department of Internal Medicine, Tongji Hospital, Tongji Medical College, Huazhong University of Science and Technology, 1095# Jiefang Ave, Wuhan, 430000 China; 2 Hubei Key Laboratory of Genetics and Molecular Mechanism of Cardiologic Disorders, Huazhong University of Science and Technology, Wuhan, 430000 China; 3 Department of Orthopedics, Union Hospital, Tongji Medical College, Huazhong University of Science and Technology, Wuhan, 430000 China

**Keywords:** Atrial fibrillation, Obesity, Epicardial fat, Inflammation, Oxidative stress, Fibrosis, Weight loss

## Abstract

The prevalence of obesity and atrial fibrillation (AF), which are inextricably linked, is rapidly increasing worldwide. Obesity rates are higher among patients with AF than healthy individuals. Some epidemiological data indicated that obese patients were more likely to develop AF, but others reported no significant correlation. Obesity-related hypertension, diabetes, and obstructive sleep apnea are all associated with AF. Additionally, increased epicardial fat, systemic inflammation, and oxidative stress caused by obesity can induce atrial enlargement, inflammatory activation, local myocardial fibrosis, and electrical conduction abnormalities, all of which led to AF and promoted its persistence. Weight loss reduced the risk and reversed natural progression of AF, which may be due to its anti-fibrosis and inflammation effect. However, fluctuations in weight offset the benefits of weight loss. Therefore, the importance of steady weight loss urges clinicians to incorporate weight management interventions in the treatment of patients with AF. In this review, we discuss the epidemiology of obesity and AF, summarize the mechanisms by which obesity triggers AF, and explain how weight loss improves the prognosis of AF.

## Introduction

Obesity has become a global epidemic. Obesity rates have nearly doubled in the last 30 years in several countries, such as China [1, 2], America [3], the United Kingdom [4], and India [5]. It is estimated that, by 2030, more than half of the world’s population will be obese, with the prevalence of severe obesity reaching 11% [6]. Truly, obesity has become a major public health concern.

Atrial fibrillation (AF) was first detected on electrocardiography by Einthoven and Lewis over 100 years ago [7]; at that time, it was considered a trivial disorder. With increased understanding of AF over the years, we have learned that AF is among the most common life-threatening arrhythmias worldwide. The current overall AF prevalence in the general population is approximately 1–2% [8–11]. Although striking, these data may underestimate the true AF incidence as patients with paroxysmal AF (pAF: defined by episodes that last < 7 days and terminate spontaneously) were not included in studies, with persistent AF (duration of episodes > 7 days) as the standard[12]. In addition, 5–35% of patients with persistent AF are asymptomatic.

There are a number of epidemiological studies that clearly demonstrate the relationship between obesity and AF (Table 1). For example, Wang TJ et al. found that regardless of gender, for every 1% increase in BMI, there was a 4% increase in AF risk in a multivariate model adjusted for cardiovascular risk factors and temporary myocardial infarction or heart failure, based on the Framingham Heart Study observation cohort of 5282 participants with an average age of 57 years and an average follow-up of 13.7 years [13]. Supportively, a prospective cohort conducted in the Danish Diets, Cancer, and Health Study by Frost L et al., enrolling 47,589 participants with an average age of 56 years, followed up for an average of 5.7 years, found that the adjusted risk ratio for AF or atrial flutter was 1.08 for males and 1.06 for females for every increase in BMI [14]. In addition, a retrospective study on middle-aged men (with an average age of 51.5 years) conducted by Rosengren A et al. revealed that excessive weight during youth and weight gain from the age of 20 to middle age were independently associated with the development of AF [15]. Similar conclusions have been confirmed in studies targeting women conducted by Tedrow UB et al. They analyzed 34,309 medical records from women’s health studies and found that overweight and obesity are associated with an increased risk of AF. Compared with participants who maintain a BMI of < 30 kg/m^2^, participants who become obese within the first 60 months from the beginning of the investigation have a 41% increased risk of developing AF [16]. Even among young fertile women with low incidence rate of AF (average age of 30.6 years), the risk ratio for AF of young obese women is 2.04, and that of extremely obese people is 3.50 comparing with normal weight women [17]. Therefore, obesity is largely associated with the increased risk of AF. Maintaining a normal weight or losing weight may prevent the onset of AF. Berkovich et al. analyzed the weight data of 18,290 men and women, and investigated the relationship between weight loss and the risk of AF. They found that weight loss was independently associated with a decrease in the risk of developing AF. For every 5 kg of weight loss, the risk of developing AF was significantly reduced by 12% [18].

Obesity is often accompanied by metabolic syndrome, diabetes, hypertension, and OSA [19]. Multiple studies evaluated the impact of these conditions as confounders on obesity-promoted AF development. In a retrospective cohort of 389,321 individuals, Lee et al. reported that metabolically healthy obesity was associated with a 20% increased risk of AF, whereas a 40% increase in the risk of AF was associated with metabolically unhealthy obesity [20]. Grundvold I et al. analyzed 7169 newly diagnosed patients with type 2 diabetes, and found that the risk of AF in overweight or obese patients at baseline was 1.9 times and 2.9 times higher than that in patients with normal BMI, respectively. 14% of patients with subsequent weight gain had a risk of AF of 1.5 times comparing with patients with stable or light weight [21]. Similarly, Kim et al. analyzed 9,797,418 patients who received the national health examination, and found that obese diabetes patients have higher risk (HR = 1.823) as compared with the diabetes patients [22]. Apart from metabolic syndrome and diabetes, Gami et al. demonstrated that obesity predicts AF independently of OSA syndrome by analyzing the factors influencing AF in 3,542 adults from Olmsted County who underwent diagnostic polysomnography between 1987 and 2003 [23]. And the recent Busselton Health Study in Busselton, Western Australia, demonstrated that a high BMI was a risk factor for AF independent of hypertension and more predictive of AF in men [24]. The above studies suggests that obesity is associated with an increased risk of AF regardless of the presence or absence of underlying diseases.

This review summarizes the AF incidence in the obese population, investigates obesity-induced AF pathogenesis and the impact of obesity on ablation, and highlights how weight loss and risk factor control improve AF prognosis.

**Table 1:** Epidemiological studies illustrating the association between obesity and AF

Adjusted risk (obesity vs. normal weight)	AF	Follow up (years)	Definition of obesity	Total	Time period	Country	Design	Study	References
HR 1.54 (male); HR 1.46 (female)	526	13.7	BMI ≥ 30 kg/m^2^	5282	1979–1999	USA	Observational cohort	Wang et al. 2004	[25]
HR 2.35 (male); HR 1.99 (female)	533	5.7	BMI ≥ 30 kg/m^2^	47,589	1993–2001	Denmark	Observational cohort	Frost et al. 2005	[14]
HR 1.36	1810	10	BMI > 30 kg/m^2^	8051	1994–2004	USA	Retrospective analysis	Zacharias et al.2005	[26]
HR 1.65	834	12.9	BMI ≥ 30 kg/m^2^	34,309	1993–2008	USA	Observational cohort	Tedrow et al. 2010	[16]
HR 2.04	110	4.6	BMI ≥ 30 kg/m^2^	271,203	2004–2009	Denmark	Observational cohort	Karasoy et al. 2013	[17]
HR 2.9	287	1.5	BMI ≥ 30 kg/m^2^	7169	1999–2009	Sweden	Observational cohort	Grundvold et al. 2015	[21]
HR 2.41	288	6	BMI ≥ 30 kg/m^2^	18,290	2000–2007	USA	Observational cohort	Berkovitch et al. 2016	[18]
HR 1.2	5106	7.5	BMI ≥ 25 kg/m^2^	389,321	2004–2006	Korea	Retrospective analysis	Lee et al. 2017	[20]
OR 1.4	1511	8	BMI > 30 kg/m^2^	67,278	2006–2013	USA	Observational cohort	Foy et al. 2018	[27]
HR 1.24	1959	4.95	BMI ≥ 25 kg/m^2^	17,134	2002–2009	Korea	Observational cohort	Lim et al. 2019	[28]
HR 1.327	196,136	8.17	BMI ≥ 30 kg/m^2^	9,797,418	2009–2017	Korea	Observational cohort	Kim et al. 2019	[22]

### AF occurrence, maintenance, and progression

AF is a complex cardiac arrhythmia that first develops in a paroxysmal form, then progresses to a persistent form, and finally continues as a long-term persistent form [29]. AF, the final outcome of different pathophysiological processes, presents with significant heterogeneity among patients [30].

AF is caused by focal ectopic-triggering activity that is mainly caused by early afterdepolarization (EAD) and delayed afterdepolarization (DAD). EAD typically occurs in the context of prolonged action potential duration (APD) [31]. In a normal action potential, L-type calcium (Ca^2+^) channels undergo voltage- and Ca^2+^-dependent inactivation, thereby limiting Ca^2+^ influx. APD progression allows L-type Ca^2+^ channels to recover from inactivation, thereby generating inward currents that lead to EAD [32]. DAD is mainly caused by abnormal sarcoplasmic reticulum (SR) Ca^2+^ leakage and diastolic SR Ca^2+^ release events. During diastole, Ca^2+^ released by SR activates the sodium–calcium exchanger, producing a transient inward current that results in membrane depolarization. In addition, gap junction coupling occurs between fibroblasts and cardiomyocytes through connexin-43 and connexin-45 proteins [33]. Compared to cardiomyocytes, the membrane potential of cardiac fibroblasts is relatively depolarized. The interaction between fibroblasts and cardiomyocytes promotes depolarization of atrial cardiomyocytes, thereby promoting DAD [34]. Any factor that leads to prolonged APD, endoplasmic reticulum (ER) Ca^2+^ leakage, ER Ca^2+^ release during diastole, or increased cardiac fibroblasts can induce ectopic-triggering activity and cause AF.

The muscular sleeve within the ostia of the pulmonary veins (PV) is the main source of ectopic-triggering activity that causes AF. The PV muscle sleeve consists of branching fibers with limited lateral coupling and abrupt fiber orientation changes, which provide a structural basis for ectopic triggering of AF [35]. Additionally, compared to cardiomyocytes in other regions of the atrium, the diastolic ER Ca^2+^ release events in the PV muscle sleeve region are increased [36], and the effective refractory period is shorter, which further increases the possibility of AF triggered by the ectopic-triggering activity in the PV muscle sleeve region.

AF maintenance and progression are based on the electrical and structural remodeling of atrial tissue. Atrial electrical remodeling is mainly caused by the following factors: downregulation of the Ca^2+^ current leading to shortening of the refractory period [37]; increased outward potassium (K^+^) current leading to accelerated repolarization and hyperpolarization of atrial cells [38]; and altered expression and localization of connexins that connect atrial myocytes, causing conduction abnormalities [39]. These changes promote reentry and maintenance of atrial activation [40]. Atrial structural remodeling mainly consists of fibrosis, atrial enlargement, and changes in cardiomyocyte ultrastructure. Changes in the cardiomyocyte ultrastructure during AF include myolysis [41], glycogen accumulation [42], gap junction impairment [43], nuclear chromatin changes [44], mitochondrial disruption [45] and redistribution, and SR alterations [46]. These changes reduce the contractility of atrial cardiomyocytes, prolong conduction, and induce and maintain AF. Atrial enlargement is an important determinant in the clinical assessment of the likelihood and prognosis of AF [47]. Greater atrial enlargement reflects a greater stretch of atrial myocytes and atrial damage. Additionally, atrial enlargement is closely associated with fibrosis [48], which can contribute to the maintenance of AF in several ways. First, fibrous tissue physically separates atrial muscle fibers, resulting in the local slowing of conduction. Second, an increased number of fibroblasts increases fibroblast–cardiomyocyte interactions, resulting in slowed conduction, cardiomyocyte depolarization, and a prolonged APD. Fibroblasts also influence the electrical activity of cardiomyocytes via paracrine bioactive substances [49]. AF typically progresses from infrequent to frequent and persistent episodes, followed by persistent AF. Clinical observations, numerous animal studies, and autopsy studies support the concept of “AF begets AF.“ In other words, AF directly induces atrial remodeling, thereby supporting AF maintenance and progression [50].

### Mechanisms of obesity-promoting AF

**Fig. 1:**
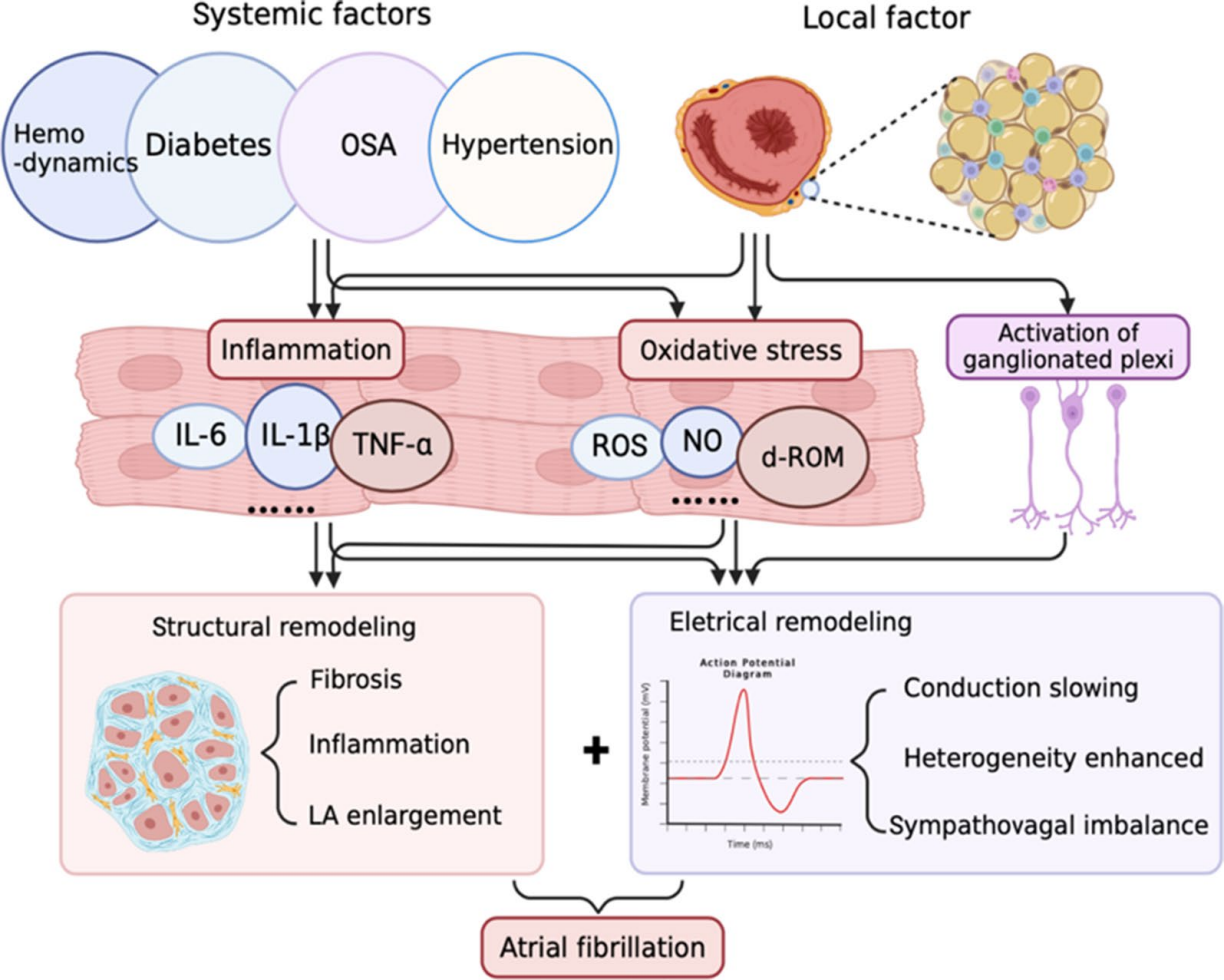
The arrhythmogenic effects of obesity on cardiomyocytes. Obesity is closely related to systemic hemodynamic changes, hypertension, sleep apnea syndrome, diabetes, and increased local epicardial fat. These systemic and local changes promote inflammation and oxidative stress, as well as activating the ganglionic plexus, leading to structural remodeling including fibrosis, left atrial enlargement, and electrical remodeling including conduction slowing and heterogeneity enhancing, and ultimately cause atrial fibrillation. *OSA* Obstructive sleep apnea, *ROS* Reactive oxygen species, *NO* nitric oxide, *d-ROM* derivative of reactive oxidative metabolites

Although epidemiological studies have established the role of obesity in independently predicting the occurrence and progression of AF, the pathophysiological mechanisms associated with AF in obese patients are complex and remain unclear. Obese patients are susceptible to AF, which may be related to systemic changes caused by obesity, such as hemodynamic changes, hypertension, diabetes, and the OSA syndrome. Additionally, in terms of molecular biology, adipose tissue secretes a variety of pro-inflammatory and pro-fibrotic factors that can accelerate the structural and functional remodeling of the left atrium and induce and maintain electrical conduction abnormalities. Oxidative stress induced by adipose tissue and activated autonomic nerves in the ganglion plexus are also involved in the occurrence of AF (Fig. 1).

### Epicardial fat in obesity

Epicardial adipose tissue (EAT) refers to adipose tissue between the visceral pericardium and epicardium [51]. The EAT and myocardium share the same microcirculation with no muscle fascia between them [52]. Thus, atrial pathophysiological changes and localized EAT cumulative infiltration are closely related [53, 54].

Clinical studies have shown that periatrial EAT is associated with AF [55, 56]. Patients with persistent AF had larger EAT volumes (EATV) and higher levels of serum inflammatory biomarkers than those with pAF, which has nothing to do with the presence of obesity, age, sex, cardiovascular diseases, diabetes, dyslipidemia or hypertension [57]. The EATV index (EATVI) [58], high left atrial EAT/total EAT ratio [59], activin A expression in the EAT [60], and EAT content in the isolated left atrial posterior wall isolation line of the posterior left atrium are all strongly associated with the increased prevalence and severity of atrial fibrillation [59, 61, 62].

Atrial electrical remodeling, inflammation, fibrosis, and neurological factors are involved in the process of EAT-promoting AF (Fig. 1). Serum levels of inflammatory markers (MCP-1, IL-1, IL-6, soluble IL-6 receptor, and TNF-α) are directly correlated with the EAT of lower density and higher volume [63]. Regional IL-1β levels in the EAT are independent risk factors for persistent AF (Fig. 1) [64]. Local conduction block and conduction delay caused by localized fibrosis of the atrium underlie the formation of re-entrant arrhythmias [65].Various pro-fibrotic cytokines/chemokines in the EAT, such as YKL-40 [66] and cTGF [67], are positively correlated with total collagen content in the left atrial myocardium (Fig. 1) [68] [69]. Blockade of the adipofibrokine (activin A) by neutralizing antibodies has been shown to reverse atrial fibrosis [70]. In addition to inducing fibroblasts, adipose tissue can also develop fibrosis [55]. The EAT is the site of the ganglionic plexus, which contains sympathetic and vagal fibers that regulate the autonomic nerves of the heart and is closely related to the initiation and maintenance of AF (Fig. 1). In addition to being pro-fibrotic and pro-inflammatory, EAT also contributes to the formation and maintenance of AF by promoting oxidative stress [71] and Ca^2+^ homeostasis imbalance [72].

### Obesity, inflammation and AF

In obese individuals, adipose tissue is infiltrated by a large number of activated macrophages, which is a state associated with systemic inflammation. The degree of macrophage infiltration is proportional to the body weight [73]. When the body weight is reduced, the number of infiltrated macrophages and inflammatory factors decrease [74, 75]. Besides, There are more CD4 + regulatory T cells involved in suppressing pro-inflammatory macrophages in lean mice [76], while obese mice are dominated by CD8 + effector T cells, which recruit and activate pro-inflammatory macrophages and promote the inflammatory cascade [77]. Therefore, the macrophages in the adipocytes of lean mice were dominated by the M2 anti-inflammatory phenotype, whereas the macrophages in the adipose tissue of obese mice were dominated by the M1 type [78].

Patients with AF had higher levels of inflammatory markers, including serum C-reactive protein (CRP), heat-shock protein (HSP) β1 (commonly referred to as HSP27), interleukin (IL)-6, IL-8, and tumor necrosis factor-α (TNF-α), when compared to patients in normal sinus rhythm [79, 80]. The pathology of atrial biopsy specimens from patients with solitary AF refractory to antiarrhythmic therapy showed lymphomonocytic infiltration and necrosis of adjacent myocytes [81], whereas these inflammatory abnormalities were absent in atrial biopsy specimens from patients in sinus rhythm. Large prospective cohort studies suggested that higher CRP levels can predict the risk of AF [82, 83], and CRP levels are reportedly higher in patients with persistent AF than in those with PAF [84]. Medicines with known anti-inflammatory effects, including glucocorticoids, N-3 fatty acids, statins, angiotensin-converting enzyme inhibitors, and angiotensin II receptor blockers, can reduce AF prevalence [85, 86]. Amiodarone’s antiarrhythmic effect may be partially dependent on its anti-inflammatory effect since it inhibits the synthesis of several cytokines, including IL-6 and TNF-α [87].

TNF-α plays a primary role in promoting atrial structural remodeling and affecting atrial ion channel function. TNF-α changes connexin-40 expression in mice and activates myofibroblasts through the transforming growth factor (TGF)-β signaling pathway, thereby inducing atrial fibrosis [88]. In addition, TNF-α can induce the expressions of matrix metalloproteinase-2 and matrix metalloproteinase-9 (MMP9), change the distribution of connexin-43 in the atrial tissue, and promote myocardial remodeling (Fig. 1) [89].The stimulation of PV cardiomyocytes with TNF-α increases the amplitude of DAD and decreases the L-type Ca^2+^ current [90]. Moreover, TNF-α reduces the Ca^2+^ content in the SR and increases the intracellular Ca^2+^ concentration during diastole, thereby inducing AF [91].

Similarly, IL-6 promotes the occurrence of AF mainly by influencing atrial electrical remodeling and fibrosis. Specifically, IL-6 induces early atrial fibrosis by activating pSTAT3/STAT3 signaling pathways[92], and promotes the expression of α-SMA, type I collagen, and type III collagen by inhibiting Tregs function [93]. IL-6 also mediates abnormalities in calcium processing in cardiac myocytes. IL-6 neutralizing antibodies can reverse the prolongation of Ca2 + transient duration and regional heterogeneity, reducing the incidence of discordant alternans, reducing the susceptibility and recurrence frequency of AF [94]. Moreover, IL-6 directly impairs the production of connexin in myocardial cells, including connexin 40 and connexin 43, thereby inducing atrial electrical remodeling and increasing the risk of AF [95].

### Obesity, oxidative stress and AF

Accumulation of oxidative stress in adipose tissue is an early event in obesity [96]. Oxidative stress is significantly increased in white adipose tissue in obese animals and humans than in non-obese subjects [97]. Weight gain and obesity in children and adolescents are positively associated with elevated oxidative stress levels [98, 99]. Moreover, maternal obesity leads to increased placental oxidative damage and placental concentrations of ROS and protein carboxyl groups [100]. Reducing body weight and fat accumulation through exercise training [101], dietary restriction [102], increasing the content of high-fiber fruits in the diet [103], or gastrectomy [104] can reduce the formation of ceruloplasmin [105], identifiable reactive oxygen metabolites, and alpha-dicarbonyl compounds; thereby, reducing oxidative stress indicators such as ROS, nitric oxide, and malondialdehyde (Fig. 1) [106].

Oxidative stress is closely related to AF development. For every 10% increase in redox glutathione, the odds of developing AF increased by 30% [107]. The increased AF odds ratios for derivatives of reactive oxidative metabolites and cysteine ​​were 6.1 and 15.9 [108], respectively. NOX-2-dependent ROS production in human right atrial samples is independently associated with postoperative AF[109]. Burst pacing–induced AF was significantly increased in transgenic mice overexpressing NOX-2, whereas the inhibition of mitochondrial oxidative stress by overexpressing mitochondrial catalase reduced the occurrence of AF [110]. Angiotensin II–induced AF is dependent on NADPH-oxidase-dependent ROS production and elevated ox-CaMKII levels [111]. Antioxidants also prevented atrial electrical remodeling in animal models of atrial tachycardia and new-onset AF after cardiac surgery. By correcting the myocardial redox balance, statins, canagliflozin, and allopurinol prevented AF-induced electrical remodeling in AF animal models and, therefore, may reduce the incidence of AF after cardiac surgery [112–114].

ROS generated by oxidative stress enhances late Na^+^ currents and induces early depolarization; thus, triggering activity [115]. Oxidative stress also induces AF by affecting L-type calcium channels and ryanodine receptor 2 (RyR2), thereby prolonging the APD [116]. Additionally, ROS stimulates the proliferation of atrial fibroblasts and promotes the expression of inflammatory and pro-fibrotic factors, such as MMP9, p38, and c-Jun (Fig. 1) [117].

### Obesity, systemic diseases and AF

Clinically, hypertension and AF often coexist [118]. More than 60% of patients with AF also have hypertension. The Framingham study showed that high blood pressure increased the risk of AF by 40-50% [119]. BMI is an important cause of high blood pressure [120]. Obese subjects were 3.5 times more likely to develop high blood pressure, and more than 60% of the cases with high blood pressure were attributable to fat accumulation [121]. Across all age groups, the prevalence of hypertension in normal-weight subjects was 34%, whereas in obese subjects, it ranged from 60 to 77% and increased accordingly with the BMI [122].Decreased myocardial systolic-diastolic function caused by hypertension eventually leads to an increase in left atrial pressure, which forces left atrial dilation and forms the basis of AF. The increase in total blood volume in obese patients leads to an increase in cardiac output, which predisposes patients to left ventricular remodeling, hypertrophy, and diastolic dysfunction. Additionally, the potential development of AF has been observed in the myocardium of hypertensive mice, such as impaired Ca^2+^ transport [123], ultrastructural changes in cardiomyocytes [124], inflammatory infiltration, and activation of fibroblasts (Fig. 1). Moreover, electrophysiological changes were found in the left atrium, such as enhanced conduction heterogeneity, shortened atrial wavelengths, and prolonged AF duration [125, 126], which was detected several weeks after the onset of hypertension.

Multiple prospective cohort studies, retrospective studies, and meta-analyses have demonstrated that diabetes is an independent risk factor for AF [127–130]. Prediabetes and diabetes increase the risk of AF by 20% and 28%, respectively, and there is a correspondence between elevated blood glucose and AF, with an 11% increased risk of AF for every 20 mg/dL increase in blood glucose [131]. After matching for age and sex, the AF risk in patients with diabetes increased to 35% [132]. With a prolonged duration of diabetes, the risk of AF further increases. Patients with diabetes also had a higher recurrence rate after catheter ablation for AF than patients without diabetes [133]. The recent Dapagliflozin Effect on Cardiovascular Events - Thrombolysis in Myocardial Infarction 58 trial suggests that dapagliflozin reduces the risk of AF/flutter events by 19% in patients with T2DM, further underscoring the role of diabetes in the induction of AF [134, 135]. However, obesity is a major contributor to the development of type 2 diabetes mellitus (T2DM) [136]. Obesity in childhood increases the risk of developing T2DM later in life. More than 80% of people diagnosed with T2DM are obese [137].

Diabetes-induced atrial fibrosis and atrial dilation underlie AF induction. Various stimuli, including inflammation, advanced glycation end products (AGEs), and TGF-β, can promote the development of fibrosis [138, 139]. Pro-inflammatory cytokines and chemokines recruit fibrotic leukocyte subsets to the interstitium. Hyperglycemia-induced accumulation of AGEs transduces fibrotic signals through the receptor for AGE pathway/oxidative stress. TGF-β/Smad signaling activates fibroblasts and induces the deposition of extracellular matrix proteins. Additionally, diabetes-induced prolongation of atrial conduction time, increased dispersion of atrial effective refractory period, and imbalance of sympathetic and parasympathetic nerve activity all increase susceptibility to AF (Fig. 1).

OSA is prevalent in obese individuals and has been identified as an important risk factor for the development and progression of AF [23, 140]. OSA increases the risk of developing AF two-fold. Interestingly, the prevalence of OSA was higher in patients with AF than in those without AF [141]. After matching for age, sex, and other electrophysiological symptoms, patients with AF had a 24% higher prevalence of OSA than those without AF [142]. In addition, OSA reduces the efficacy of antiarrhythmic drugs, electrical cardioversion, and catheter ablation in AF treatment [143].

The relationship between OSA syndrome and AF is complex. Multiple studies have confirmed that OSA is an independent predictor of myocardial diastolic dysfunction, which may lead to left atrial enlargement [144, 145]. Hypoxic episodes caused by OSA activate the sympathetic nerves, leading to tachycardia and increased blood pressure, resulting in relative ischemia of the atrial myocardium [146, 147]. Furthermore, hypoxia reduces atrial conduction velocity and increases its heterogeneity, thus increasing susceptibility to AF [148, 149]. Prolonged apnea also promotes neuronal firing in the ganglionic plexus, near the PV [150]. Additionally, OSA-induced sympathovagal imbalance and atrial fibrosis promote the occurrence and maintenance of AF (Fig. 1) [151].

### Effect of weight loss on AF

**Fig. 2:**
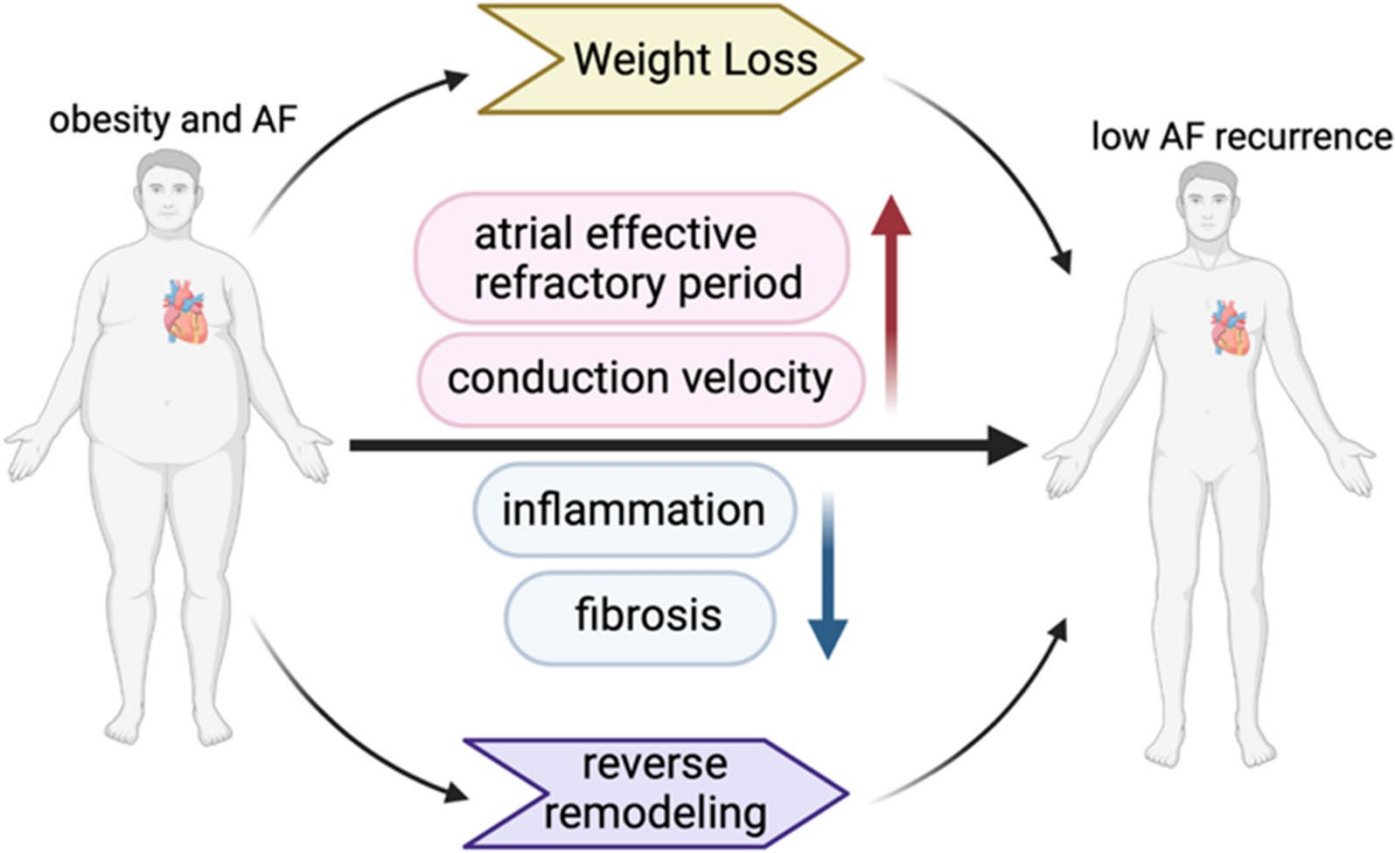
Proposed mechanism of benefits of weight loss to reduce AF. Reverse remodeling of cardiac structure and electrophysiology occurs after weight loss, manifested as decreased inflammation and fibrosis, increased atrial effective refractory period, and increased conduction velocity

Multiple studies have shown that obesity management reverses the natural progression of AF [152–154]. In the Swedish obese subject study conducted in Sweden, bariatric surgery was associated with sustained weight loss (18% weight loss after 20 years), and in the 19-year follow-up, the risk of AF was reduced by 29% in the bariatric surgery group than it was in the control group (Fig. 2) [25]. Donnellan et al. compared the effects of different bariatric surgeries on AF, and the results showed that the percentage of weight loss significantly correlated with the reversal of AF [155]. Weight loss in obese patients with long-term persistent AF also effectively improved their quality of life, although there was no change in symptom severity or long-term ablation outcomes [156]. Mahajan et al. partially explained the role of weight loss in the development of AF in animal experiments conducted on 30 sheep. After weight loss in obese sheep, the cardiac structure and electrophysiology are inversely reconstructed, which manifest as a reduction in inflammation and fibrosis and an increase in the atrial effective refractory period and conduction velocity (Fig. 2) [157].

Sustained and stable weight loss is an essential factor in obesity management to reverse AF, and weight fluctuations offset the benefits of weight loss. The Long-Term Effect of Goal Directed Weight Management on Atrial Fibrillation Cohort: A 5 Year follow-up study showed that when the weight loss was ≥ 10%, the arrhythmic free survival rate, AF burden, and symptom severity were significantly decreased, while weight fluctuation > 5% partially offset this benefit, and the risk of arrhythmia recurrence was doubled [158]. A meta-analysis conducted by Jones et al. showed that weight loss of 5% was not associated with a significant change in AF incidence (HR 1.04) [159]. Johansson et al. conducted a health survey of more than 100,000 people in northern Sweden and found that middle-aged weight loss was not significantly correlated with AF risk, which may be related to fluctuations in body weight [160]. Lee et al. quantified the effects of weight variability on AF. They found that for every 1 standard deviation increase in weight variability, the risk of AF increased by 5%. Except for the extremely obese group (BMI ≥ 30 kg/m^2^), high body weight variability in all baseline BMI groups was significantly associated with AF occurrence, and this correlation was stronger in subjects with lower body weight [161].

The current mainstream view is that it is beneficial to reduce weight and manage the impact of risk factors on the prognosis of AF [19]. The American Heart Association strongly recommends reducing AF by adjusting lifestyle and controlling risk factors, including obesity, lack of exercise, OSA, diabetes, hypertension, and other modifiable factors [162]. However, a multicenter cross-sectional descriptive study conducted in Belgium found that more than 30% of obese patients did not understand the positive impact of weight loss on AF progression. Patients with low education, hypertension, who are living alone, who have never tried to lose weight, and have low BMI, but are still at high risk of developing AF, lacked the intrinsic motivation to lose weight [163]. Weight management is an effective intervention that does not require marketing and a large amount of financial promotion, such as drug- and device-based therapies, and should be vigorously promoted. Cardiovascular physicians should also include weight management programs for the treatment of obese patients with AF to improve patient-centered treatment outcomes [164].

## Conclusion and perspective remarks

Obesity is a major risk factor for AF. Obese individuals are more likely to develop AF than normal-weight individuals. Adipose tissue leads to left atrial enlargement and electrical remodeling through various mechanisms, including inflammation and oxidative stress. This induces AF development and promotes type switching. Weight loss reduces AF development and is associated with a reduced rate of AF recurrence after ablation. Although the relationship between obesity and AF has been well explained, the pathophysiological mechanisms are complex and have not been fully elucidated. For example, from the perspective of metabolism, obesity is categorized into metabolically healthy and unhealthy. Even if metabolically healthy obese patients have a high BMI, the AF risk is low; thus, using BMI alone to identify obesity is not accurate. In addition, adipose tissue is classified as white, beige, and brown fats, which are distributed throughout the body and function differently. Do these different changes in fat correspond to different AF risks? Is it necessary to define the role of fat type in AF? Obesity is a risk factor for the recurrence of PAF after catheter ablation, but not for permanent AF; therefore, further studies are needed to explore the differences in the development of paroxysmal and permanent AF.

Studies have demonstrated the effect of obesity on AF, with obesity in adults as the main target of mechanistic exploration; however, obesity in early life is a better predictor of AF risk throughout adulthood. Further studies are required to explore the mechanisms by which early obesity promotes AF development in adulthood.

Epicardial fat in obese patients plays a key role in left atrial structural and electrical remodeling. The size and thickness of epicardial tissue predict the occurrence, development, and type switching of AF. However, the current imaging techniques used to detect EAT cannot meet the requirements of speed, economy, and accuracy at the same time, and cannot quantify the relationship between epicardial fat volume and AF severity and prognosis. Understanding the importance of EAT in AF development is still in the clinical research stage. Exploration of the possibility of linking EAT with AF diagnosis and treatment may be an important direction in future research.

The current mainstream view is that weight loss is beneficial in reducing the occurrence of AF; however, there is still an “obesity paradox.“ Specifically, weight loss was not associated with a reduced AF risk [165], whereas an increased BMI was independently associated with a reduced risk of stroke and improved survival after AF [166]. Therefore, to clarify the status of weight loss in AF prevention and treatment, it is necessary to explore the reasons for the obesity paradox. The idea that weight loss can reduce the AF burden has not been widely accepted, and most people lack intrinsic motivation to lose weight. Therefore, it is necessary to vigorously promote weight management and make weight management programs mandatory for patients with AF as a part of their daily treatment.

## Data Availability

Not applicable.

## Abbreviations

AF: Atrial fibrillation
AGE: Advanced glycation end product
Angptl2: Angiopoietin-like protein 2
ATPase: Adenosine triphosphatase
BMI: Body mass index
CRP: C-reactive protein
DAD: Delayed afterdepolarization
DIO: Diet-induced obese
EAD: Early afterdepolarization
EAT: Epicardial adipose tissue
EATV: EAT volumes
EATVI: EATV index
ER: Endoplasmic reticulum
HFD: High-fat diet
HR: Hazard ratio
HSP: Heat-shock protein
LV: Left ventricular
MCP-1: Monocyte chemoattractant protein 1
MMP9: Matrix metalloproteinase-9
NADPH: Nicotinamide adenine dinucleotide phosphate
NE: Norepinephrine
NOX: NADPH-oxidase
OSA: Obstructive sleep apnea
PAF: Paroxysmal AF
PAT: Paroxysmal atrial tachycardia
PKA: Protein kinase A
PV: Pulmonary veins
ROS: Reactive oxygen species
RyR2: Ryanodine receptor 2
SR: Sarcoplasmic reticulum
T2DM: Type 2 diabetes mellitus
TNF: Tumor necrosis factor
